# Elimination of Cefquinome Sulfate Residue in Cow’s Milk after Intrauterine Infusion

**DOI:** 10.3390/metabo13040492

**Published:** 2023-03-29

**Authors:** Chunshuang Liu, Mingyue Han, Honglei Wang, Xiaojie Chen, Yaoxin Tang, Daokang Zhang, Xiubo Li, Yiming Liu

**Affiliations:** 1 National Feed Drug Reference Laboratories, Feed Research Institute, Chinese Academy of Agricultural Sciences, Beijing 100081, China; 2 Key Laboratory of Animal Antimicrobial Resistance Surveillance, Ministry of Agriculture and Rural Affairs, Feed Research Institute, Chinese Academy of Agricultural Sciences, Beijing 100081, China; 3 Laboratory of Quality & Safety Risk Assessment for Products on Feed-origin Risk Factor Ministry of Agriculture and Rural Affairs, Feed Research Institute, Chinese Academy of Agricultural Sciences, Beijing 100081, China; 4 Waters Technology Co., Ltd., Shanghai 201206, China

**Keywords:** cefquinome sulfate intrauterine infusion, cow, milk, residue, UPLC-MS/MS

## Abstract

As set in the maximum residue limit regulations of the European Commission, this study aimed to obtain the residual parameters in milk with optimized UPLC-MS/MS conditions and to determine the conclusive drug withdrawal period to ensure food safety. In this research, an ultra-high performance liquid chromatography-tandem mass spectrometry (UPLC-MS/MS) method was developed to study cefquinome sulfate’s residue elimination in milk and to calculate cefquinome’s withdrawal period. Twelve healthy cows free of endometritis were selected for the experiment. Before using the drug, the vaginal orifice and perineum of each cow was disinfected. One dose of intrauterine perfusion was used for each cow, followed by an additional dose after 72 h. Before administration and 12 h, 18 h, 24 h, 36 h, 42 h, 48 h, 60 h, 66 h, 72 h, 84 h, 90 h, and 96 h after the last dose, milk (10 mL) was gathered from each cow’s teat and pooled. For the measurement of cefquinome concentrations in milk, UPLC-MS/MS was performed. A calibration curve was generated using linear regression as follows: Y = 250.86X − 102.29, with a correlation coefficient of 0.9996; the limits of detection and the limits of quantitation were 0.1 μg·kg^−1^ and 0.2 μg·kg^−1^, respectively. The average recovery of cefquinome was 88.60 ± 16.33% at 0.2 μg·kg^−1^, 100.95 ± 2.54% at 10 μg·kg^−1^, and 97.29 ± 1.77% at 50 μg·kg^−1^. For 5 consecutive days at the three spiking levels, the intra and inter-day relative standard deviations (RSD) were 1.28%–13.73% and 1.81%–18.44%, respectively; the residual amount of cefquinome was less than the maximum residue limit of 20 μg·kg^−1^, 36 h after administration; and the residual amount was less than the limit of detection (0.1 μg·kg^−1^) 48 h after administration. The withdrawal time of cefquinome in cow’s milk was 39.8 h, as calculated using WTM1.4 software. In terms of clinical practical use, the withdrawal period of milk was temporarily set at 48 h after the administration of the cefquinome sulfate uterus injection to cows, in accordance with the recommended dose and course.

## 1. Introduction

The uterine cavity of cattle is often polluted with environmental bacteria postpartum [1,2]. Uterine diseases such as endometritis and metritis continue to occur in high-producing dairy cows [3]. Endometritis may result in decreased fertility and economic losses in dairy cows. Recent studies have focused on the development of effective treatment schemes for endometritis. For 3–4 weeks after delivery, the uterine cavity is continuously polluted, exculpated, and re-contaminated with bacteria. In addition, 10–15% of cattle develop clinical signs of uterine infection and can cause considerable infertility, even after symptoms resolve [4,5]. Studies have shown that the treatment effect of antibiotics in cows for endometritis [6] was superior to or equivalent to that of prostaglandin treatment [7]. Many of the antibiotics that were selected for this research have been used to treat uterine infections in cattle. To avoid treating sick animals with potentially ineffective compounds, in vivo susceptibility testing often helps predict the clinical outcome of antimicrobial therapy and allows clinical research to focus on antibiotics that are likely to be successful in vivo. Studies have shown that certain cephalosporins may be more suitable for the treatment of metritis and endometritis in 38% of cases associated with *Escherichia coli*. [8]. One approach to reduce the influence of uterine disorders on fertility is intrauterine (I.U.) treatment with antibiotics. Cephapirin benzathine intrauterine infusion, a first-generation cephalosporin, has improved the reproductive performance of cows with subclinical endometritis or clinical endometritis [9].

Cefquinome has been approved for animal usage in many countries among fourth-generation cephalosporins [10,11]. Cefquinome has been approved by the European Commission for Veterinary Medicines for the treatment of severe bacterial infections; it is highly stable in the presence of lactamase [12,13], and has excellent pharmacokinetic characteristics [14,15,16,17]. Cefquinome was developed by Hoechst AG in Germany in the 1980s. It was listed in Germany and the UK in 1993 and 1994 under the trade names Cobactan and Cephguard, respectively. The common dosage forms of cefquinome sulfate formulations are injection, breast injection, lyophilized powder injection, and cream. In terms of bacterial infections in livestock and poultry, cefquinome sulfate was widely used in clinical treatment. Studies have shown that cefquinome is effective for the treatment of animal diseases such as mastitis in dairy cows [18,19], severe pneumonia in piglets [20], and bacterial infections and septicemia in horses [21,22]. The cefquinome is potent against the bacteria that cause clinical mastitis in dairy cows [23], and it has been effectively utilized for the treatment and prevention of cattle endometritis [24].

Global concern has been raised by the problem of veterinary drug residues in animal-derived foods in recent years. Veterinary drug residues can severely impact human health and safety. There are currently several methods for the determination of cefquinome in milk and animal tissues [25,26,27,28]. In our study, an UPLC-MS/MS method was developed for studying the elimination of cefquinome sulfate intrauterine infusion residues in milk, which was conducted to determine the withdrawal period of cefquinome sulfate intrauterine infusion and to provide a scientific basis for the treatment and production of dairy cows.

## 2. Materials and Methods

### 2.1. Instruments and Reagents

Cefquinome sulfate reference standard (80.1%, batch number: K0320906) was purchased from the China Institute of Veterinary Drug Control (Beijing, China). Cefquinome sulfate intrauterine infusion (containing 900 mg cefquinome in 25 mL) was supplied by Huaqinyuan Animal Pharmaceutical Co., Ltd. (Beijing, China). Acetonitrile and methanol (mass spectrometry-grade) were obtained from Thermo Fisher Scientific (Fair Lawn, NJ, USA). Ultrapure water 99 was prepared by PALL (Cascada I PALL, New York, NY, USA). Other chemical substances and reagents applied in this experimentation were of analytic grade (Beijing, China).

### 2.2. Sample Collection

Twelve healthy cows free of endometritis were selected for the experiment. The farmed animals were managed according to the procedures of the farm’s feeding. Before using the drug, the vaginal orifice and perineum of each cow was disinfected. One dose of intrauterine perfusion was used for each cow, followed by an additional dose after 72 h. Before administration and 12 h, 18 h, 24 h, 36 h, 42 h, 48 h, 60 h, 66 h, 72 h, 84 h, 90 h, and 96 h after the last dose, milk (10 mL) was gathered from each cow’s teat and pooled. The tubes containing the milk mixture were then stored at–40 °C until analysis. All animal experimentation procedures were agreed by the Animals Use and Feed Research Institute’s Care Committee, Chinese Academy of Agricultural Sciences (number: FRI-CAAS-20130511) and worked in conformity to its regulations.

### 2.3. Establishment of a UPLC-MS/MS Assay

#### 2.3.1. UPLC Conditions

The UPLC-MS/MS system was equipped with an ACQUITY ultra-high performance liquid chromatography (UPLC) system (Waters Corporation, Milford, USA). The UPLC system was separated on a UPLC BEH C18 (50 mm × 2.1 mm, 1.7 μm) column (Temperature: 40 °C ). Acetonitrile (solution A) and 0.1% (*v*/*v*) formic acid in water (solution B) were selected as mobile phases for a created gradient elution. Gradient elution was performed at 5 µL injection volume and a flow rate of 0.35 mL/min for a total run time of 6 min. The specific gradient elution process is as follows: first, B runs at 95% for 0.8 min; second, linearly reduced from 95% B to 50% B for 0.8 to 3 min; third, linearly reducing from 50% B to 10% B for 4 to 5 min; and, finally, increasing linearity from 10% B to 95% B for 5.01 to 6 min.

#### 2.3.2. Mass Spectrometry Conditions

The UPLC-MS/MS system was equipped with a Xevo TQ-S triple quadrupole mass spectrometer (Waters company, Milford, MA, USA). The parameters for using the positive ionization mode (ESI+) were as follows: capillary voltage, 2.0 kV; atomization temperature, 150 °C; desolvation gas flow, 700 L·hr^−1^; cone-hole blowback air flow, 30 L·hr^−1^; and secondary collision gas, argon gas. The cefquinome sulfate multiple reaction monitoring parameters are listed in Table 1.

### 2.4. Sample Pretreatment

#### Extraction and Purification

Approximately 3 mL of acetonitrile was spiked to a 15 mL tube with 1 mL of a milk sample after vortexing the mixture for 1 min and then centrifuging it at 7104× *g* for 10 min. The supernatant liquid was added into a 10 mL tube, and 2 mL of acetonitrile was taken to extract the remaining residue again. The two supernatants were combined in the same 10 mL test tube and concentrated to dryness at 40 °C under nitrogen. 3 mL of water was taken to dissolve the residues, and after treating the solid phase extraction cartridge (SPE) with 3 mL of methanol and 3 mL water, the mixture was added to the SPE cartridge. The solution in the SPE cartridge percolates through by gravity. The cartridge was then washed with 3 mL of methanol water with a concentration of 5%, the analyte was eluted from the SPE cartridge with 1.5 mL methanol, and the eluent was added to a 10 mL tube and concentrated to dryness by nitrogen blowing at 40 °C in a water bath. The residue was reconstituted in 1 mL formic acid and acetonitrile (*v*/*v*, 5:95) aqueous solution, and filtered by using a 0.22-µm nylon syringe filter in the front of analysis through UPLC-MS/MS.

### 2.5. Method Validation

The analytical methods developed in this experiment follow the European Commission Guidelines 2002/657/2002 [29]. A series of matrix-matched standard solutions were prepared by adding cefquinome standards at concentrations of 0.2, 1, 5, 10, 25, and 50 μg·kg^−1^ to the blank matrix (milk). The standard calibration curve was generated using the cefquinome peak area vs. the standard cefquinome concentration. The limit of detection (LOD) and the limit of quantitation (LOQ) were defined by adding a known concentration of cefquinome into the blank milk samples, and the minimum concentrations came across the demands of signal-to-noise ratios of ≥3 and ≥10, respectively. The accuracy and precision were determined via the 1 mL blank milk spiked with three concentrations (0.2, 10, and 50 μg·kg^−1^) in five replicates per concentration on the same day for 5 successive days. Precision was evaluated by spiked recovery ± standard deviation (SD), and accuracy was evaluated by relative standard deviations (RSDs). It has been reported that the presence of analytes matrix effects (ME) can be quantified using the modification of the equation [30]. The peak area of analyte that was made through the matrix-matched solution (25 μg·kg^−1^) was compared with the peak area of analyte that was prepared in the mobile phase solution (25 μg·kg^−1^) for evaluating the developed method’s matrix effects. The matrix effect (ME) was calculated using the following formula: 

ME = (Peak area of analyte in matrix matching solution/Peak area of analyte in mobile phase solution × 100%) − 100%.

When the value of ME is between −20% and 20%, it shows a weak matrix effect. When the value of ME < −20% or the value of ME > 20%, it shows a strong matrix effect [31].

## 3. Results and Discussion

### 3.1. Optimization of UPLC-MS/MS Analysis Conditions

**Figure 1:**
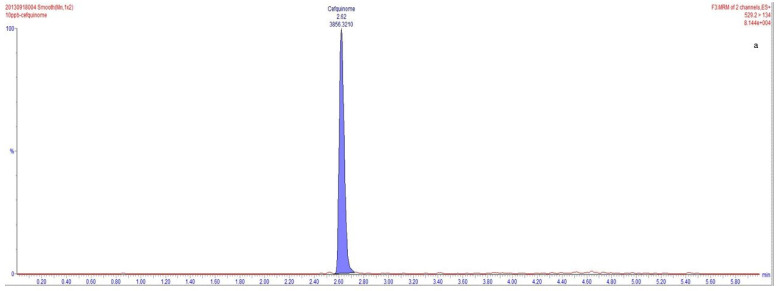

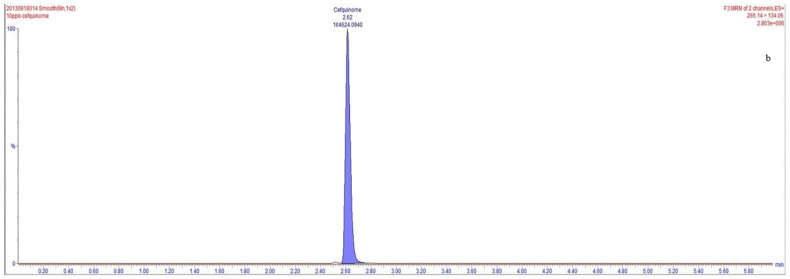
Chromatograms comparison of different ionization modes. (**a**,**b**), Blank milk matrix spiked with cefquinome sulfate (10 μg·kg^–1^).

The full scan spectrum was obtained to screen out the most abundant *m/z* value of cefquinome. Applying the ESI source in the positive ionization mode is the widespread method for analyzing cefquinome. In several studies on cefquinome detection, [M+H]^+^ ions account for more––the precursor ion *m/z* 529.1, the product ion *m/z* 134.1, and the product ion *m/z* 396 [32,33]. However, in our study, [M + 2H]^2+^ ions account for more––and the precursor ion *m/z* 265.1, the product ion *m/z* 134.1, and the product ion *m/z* 199.3. Therefore, these ions were selected for the detection of cefquinome in milk. In this study, different precursor ion (*m/z* 265.1 and *m/z* 529.1) and the same product ion (*m/z* 134.1) were selected to detect the same concentration of cefquinome. The results showed that the response value of 2.803 × 10^6^ during the fragmentation from *m/z* 265.1 precursor ion to product ion *m/z* 134.1 was significantly higher than that of the response value of 8.144 × 10^4^ during the fragmentation from *m/z* 529.1 precursor ion to product ion *m/z* 134.1. The peak area of 104,624.0940 during *m/z* 265.1 precursor ion was fragmented to product ion *m/z* 134.1, and was significantly higher than that of the peak area of 3856.3210 during *m/z* 529.1 precursor ion, which was fragmented to product ion *m/z* 134.1 (Figure 1). After optimization of the mass spectrometry parameters, we identified the precursor ion *m/z* 265.1, the product ion *m/z* 134.1, and the product ion *m/z* 199.3 for the detection of cefquinome in milk.

### 3.2. Method Validation

The performance values of the established UPLC-MS/MS analytical method assay (Table 2) meet the criteria established in decision 2002/657/EC [29].

A linear range of 0.2–50 μg·kg^−1^ was used, and the concentrations of the preparations were well linearized regarding the peak area. A calibration curve was generated using linear regression as follows: Y = 250.86X − 102.29, with a correlation coefficient of 0.9996 (Table 3).

The sensitivity of the method is often determined by measuring the LOD and LOQ of the analyte. LOD was based on a chromatographic peak with a signal-to-noise ratio of 3, and LOQ was based on a chromatographic peak with a signal-to-noise ratio of 10. In this experiment, the LOD and the LOQ were 0.1 μg·kg^−1^ and 0.2 μg·kg^−1^ (Table 3), respectively, which were lower than those of other studies [34,35], indicating that our method has a good detection performance.

The accuracy and precision for determination of cefquinome at three spiking concentrations (0.2, 10, and 50 μg·kg^−1^) are shown in Table 2. Method accuracy was assessed in sessions of cefquinome mean recovery at the 3 concentrations. The average recovery of cefquinome was 88.60 ± 16.33% at 0.2 μg·kg^−1^, 100.95 ± 2.54% at 10 μg·kg^−1^, and 97.29 ± 1.77% at 50 μg·kg^−1^. The average recovery of cefquinome was 100.95 ± 2.54% at 10 μg·kg^−1^. The acceptance standards for mean recovery at each concentration grade vary. For concentrations ≥ 10 μg·kg^−1^, the recovery deviation must be within the range of −20% to +10% [29]. Average recoveries for all three concentrations were thought to be acceptable (Table 2). Precision was assessed by repeatability and reproducibility at the three adding levels. For 5 consecutive days at the three spiking levels, the intra-day and inter-day RSDs were 1.28–13.73% and 1.81–18.44%, respectively. At one instance, the inter-day RSD was >15%. Nevertheless, in most instances, the RSD was <15%. The results show that the repeatability, reproducibility, and recovery of the method proposed in this study are better than that reported in other related studies using equal spiking concentrations [32,35].

**Figure 2:**
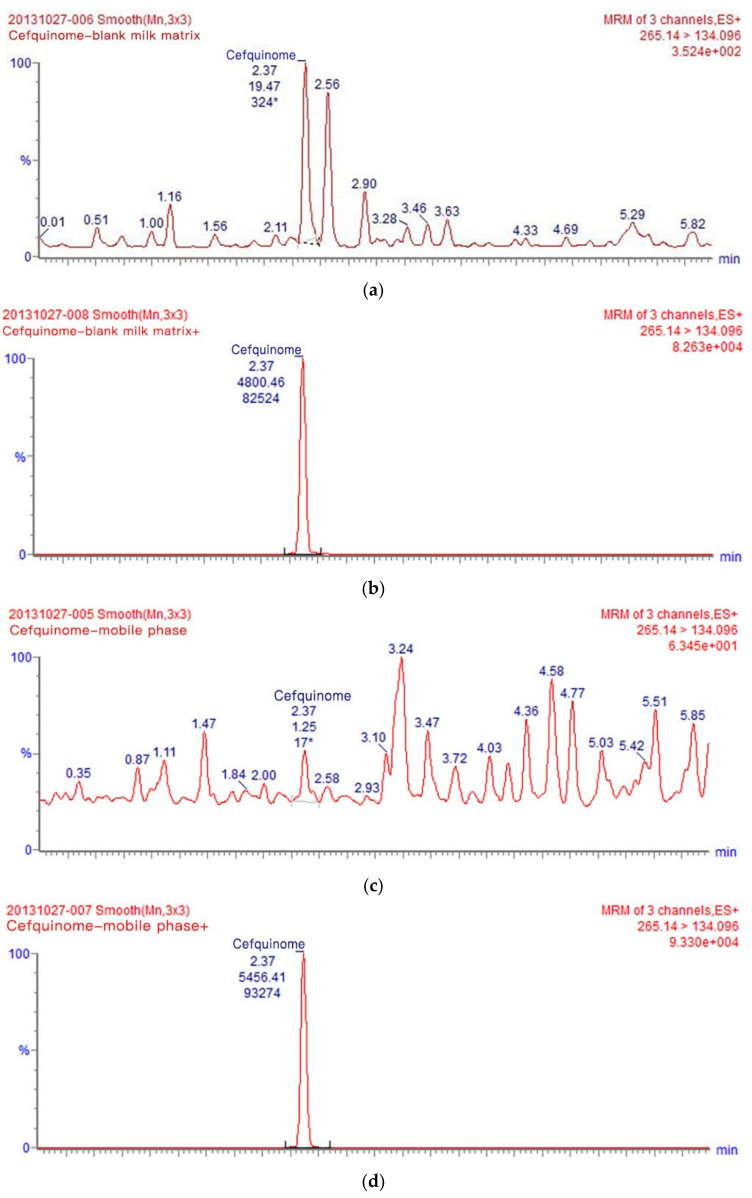
The chromatograms of the UPLC-MS/MS validation method. (**a**) Blank milk matrix sample. (**b**) Blank milk matrix sample added with cefquinome sulfate (25 μg·kg^–1^). (**c**) Blank mobile phase sample. (**d**) Blank mobile phase added with cefquinome sulfate (25 μg·kg^–1^). * Ionic abundance.

In many studies, before developing a suitable analytical method, it is necessary to comprehensively consider that milk contains lipids, proteins, minerals, and other substances that may interfere with the detection of cefquinome [36,37]. The compounds’ quantitative analysis is compromised by an endogenous origin substance in LC-MS with an electro spray ionization source that can severely influence it at trace levels, and can thus influence the method accuracy greatly. To assess the effect of the cow’s milk matrix on cefquinome, chromatograms of blank milk matrix sample supplemented with cefquinome (25 μg·kg^−1^) were compared with chromatograms of blank mobile phase sample spiked with cefquinome (25 μg·kg^−1^), reconstituted in the same mobile phase (Figure 2). When the value of ME is between −20% and 20%, it shows a weak matrix effect. When the value of ME is <−20% or the value of ME > 20%, it shows a strong matrix effect [30,38,39]. Throughout detection, undetected matrix components may have a weak or strong matrix effect strength on the analyte, and influence the accuracy and the repeatability of the analysis. In our study, ME was calculated as −12%, −12% was between −20% and 20%, and a weak matrix effect was produced when cefquinome was detected in milk (Figure 2). The obtained matrix effect results show that calibration curves using a matrix matching calibration curve play an important role in complex cow’s milk matrices to determine cefquinome content. Matrix interferences that are detected in cow’s milk can be minimized utilizing matrix-matched calibration standards. These results suggest that this method is appropriate for detecting cefquinome residues in cow’s milk samples.

### 3.3. Application to Real Samples

We analyzed the residual changes in cefquinome in milk after administration of the same dose to 12 healthy cows without endometritis to demonstrate the withdrawal period of cefquinome. All milk samples were treated by a pretreatment method and the concentration of cefquinome in milk was detected by UPLC-MS/MS. The residue concentrations of cefquinome sulfate in milk samples from 12 cows at various time points between 12 h and 96 h after dosing are shown in Table 4.

The results showed that the average residual concentrations in the corresponding 12 milk samples at each time point were as follows: 178.74 ± 74.66 μg·kg^−1^ for 12 h, 101.26 ± 14.77 μg·kg^−1^ for 18 h, 32.19 ± 14.47 μg·kg^−1^ for 24 h, 7.14 ± 2.78 μg·kg^−1^ for 36 h, and 3.00 ± 2.07 μg·kg^−1^ for 42 h, and all residual concentrations were lower than 0.1 μg·kg^−1^ from 48 h to 96 h. In addition, the results showed that the residual concentration of cefquinome sulfate in milk from the first milking (12 h) after administration was between 104.22 μg·kg^−1^ and 347.76 μg·kg^−1^. The level of cefquinome sulfate in the milk rapidly decreased to a range of 10.43–52.84 μg·kg^−1^ after 24 h. Cefquinome residues were already less than the maximum residue limit (MRL) of 20 μg·kg^−1^ in milk samples that were collected 36 h after the last dose. In the milk sample collected 48 h after the last dose, the concentration of cefquinome was below the detection limit of this method (0.1 μg·kg^−1^). In terms of clinical practical use, the withdrawal period of milk was temporarily set as 48 h after the administration of the cefquinome sulfate uterus injection to cows in accordance with the recommended dose and course.

### 3.4. Withdrawal Period

Antibiotics are widely applied in animals for dissimilar aims, including feed efficiency and increment promotion as well as preventative and therapeutic impacts. At least 60,000 tons of antibiotics are applied every year in the livestock industry [40,41]. Metabolites of antibiotics are mostly excreted in the urine and a small amount in feces. A certain proportion of the drugs, nevertheless, may remain in animal foods [40]. The dairy production process can be disrupted by antibiotic residues in raw milk while also leading to gastrointestinal issues and allergic responses in consumers [42]. A study of 973 milk samples that were collected across the Netherlands that used a microbial multiplex system identified nine samples positive for β-lactam antibiotic residues [43]. A screening study of 127 raw milk samples that were gathered from dairy farms in Kosovo demonstrated that β-lactam drugs were detectable in 50.4% of the samples [44,45]. In a study of local and imported milk samples from Kuwait, 28 of the 313 imported pasteurized milk samples were discovered to be higher than the MRL [46]. With the worldwide emphasis on milk safety and quality, β-lactam detection methods have also been diversified. To prevent cefquinome residues in milk from posing health risks to consumers, the maximum residue limit (MRL) set by the European Commission is 20 μg·kg^−1^ [47]. It is necessary to investigate whether the length of the drug withdrawal period of cows that are treated with cefquinome influences residual depletion features.

The interval between the last dose of a veterinary medicinal product to animals and the production of foodstuff was called the withdrawal period. WTM 1.4 was published by the European Medicinal Products Evaluation Agency (EMEA), which is based in London, UK, to calculate the withdrawal times in milk using the statistical method proposed by the Committee for Veterinary Medicinal Products (CVMP). For studying the elimination of cefquinome residue in milk, WTM 1.4 software was used to calculate the milk withdrawal period of cefquinome, and it was found to be 39.8 h.

The withdrawal period may vary with antibiotics, and the withdrawal period ranged from a few days to a couple of weeks. β-lactam antibiotics are widely used in the treatment of dairy cow diseases. At present, there are many studies on the withdrawal period of β-lactam antibiotics in milk. Smith et al. [48]. reported that 300 mg ceftiofur was injected into the mammary gland quarters of each cow twice at 12 h intervals, and it was found that the withdrawal period of ceftiofur in milk was 168 h after the intramammary administration. Sato et al. [49]. used an intramammary infusion of cefazolin sodium (CEZ) into the teats of cows with lactation difficulties. The results showed that the total time from the cessation of milking to the resumption of milking at 72 h after milking was the withdrawal period of cefazolin sodium. Zeng et al. [50]. studied 20 goats by intramuscular injection of penicillin G (2 mL of drug preparation) and intramammary infusion of cephapirin sodium (cephalosporin activity 200 mg). It was found that the withdrawal period of penicillin G and cephapirin sodium was 72 h and 120 h, respectively, as seen by Stockler et al. [36]. After the infusion of cephapirin into lactating cows, the withdrawal period of cephapirin was 96 h. In our study, the withdrawal period of cefquinome sulfate intrauterine infusion was 39.8 h. In conclusion, after 39.8 h of cefquinome sulfate intrauterine infusion, the residual amount of cefquinome in milk was lower than the MRL prescribed by the European Union.

When the correct withdrawal periods are known, improper drug use and the incidence of antibiotic resistance [51] can be reduced [37,52]. In effect, β-lactam residues can foster drug resistance [53,54] and, if imbibed at certain concentrations, may lead to adverse effects in humans [55]. Because pediatricians recommend cow’s milk for infants and children, it is important to consider potential intolerances or allergies to cow’s milk. A new generation of cephalosporins has been widely used in humans and animals. If cephalosporins are used and bacteria develop resistance, it may severely threaten human health. Therefore, prudent and reasonable application of cephalosporins in food animals and reasonable optimization of an antibiotic dosage plan are crucial to inhibiting the production of drug-resistant bacteria, which not only ensures a therapeutic effect but also minimizes the emergence of drug resistance in pathogens [56].

## 4. Conclusions

In this study, UPLC-MS/MS conditions and a preprocessing method were established and validated for detecting cefquinome residue in cow’s milk. The cefquinome recovery was between 72.27% and 103.21%. The UPLC-MS/MS method and a processing method were established for cefquinome’s detection in the milk of cows. The results show that the verification parameters of the method all meet the criteria of commission decision 2002/657/EC [29]. After cows underwent cefquinome sulfate infusion, the established method was used to study cefquinome’s elimination in milk. Approximately 36 h after administration, cefquinome residue concentration was below the MRL of 20 μg·kg^−1^ in milk samples that were collected. The cefquinome residue concentration in samples collected 48 h after administration was lower than the method’s limit of detection. The withdrawal period of cefquinome in milk was 39.8 h. In terms of clinical practical use, the withdrawal period of milk was temporarily set at 48 h after the administration of cefquinome sulfate uterus injection of cows in accordance with the recommended dose and course. The results of cefquinome residue in milk provide reference for the rational use of cefquinome residue and milk food safety.

## Figures and Tables

**Table 1:** Cefquinome sulfate mass spectrometry parameters.

Precursorion (*m*/*z*)	Production (*m/z*)	Cone Voltage (V)	Collision Energy (eV)
265.1	134.1 *	28	16
265.1	199.3	28	14

* Qualitative ion.

**Table 2:** Validation data for cefquinome determination in milk at three added concentrations.

Spiking Concentration (μg·kg^−1^)	Recovery Rate (%)	SD (%)	Intra-Day RSD (%)					Inter-Day RSD (%)
			Day 1	Day 2	Day 3	Day 4	Day 5	
50	97.29	1.77	1.67	1.56	1.35	1.28	1.52	1.81
10	100.95	2.54	2.26	2.22	1.69	1.97	3.45	2.51
0.2	88.60	16.33	12.92	13.00	10.50	13.73	12.29	18.44

**Table 3:** The calibration equation, correlation coefficient, LOD, and LOQ for cefquinome in milk.

Name	Content
Calibration Equation	Y = 250.86X − 102.29
A linear range (μg·kg^−1^)	0.2–50
Correlation coefficient (R^2^)	0.9996
The limits of detection (LOD) (μg·kg^−1^)	0.1
The limits of quantitation (LOQ) (μg·kg^−1^)	0.2

**Table 4:** Residues’ concentrations of cefquinome sulfate in samples collected from 12 cows.

Time (h)	Concentration of Cefquinome in Milk (μg·kg^−1^)													
	Cow #1	Cow #2	Cow #3	Cow #4	Cow #5	Cow #6	Cow #7	Cow #8	Cow #9	Cow #10	Cow #11	Cow #12	Average	SD
12	347.76	197.37	260.08	106.18	204.30	139.31	117.15	192.52	104.22	115.81	229.16	131.00	178.74	74.66
18	115.53	73.29	82.20	66.93	85.46	70.01	74.99	100.85	81.37	63.39	78.02	89.06	101.26	14.77
24	45.31	35.48	47.22	35.65	43.75	21.67	14.26	33.53	34.21	10.43	52.84	11.96	32.19	14.47
36	9.70	9.46	9.25	6.52	4.90	9.25	3.83	3.11	7.01	2.96	9.64	9.99	7.14	2.78
42	7.74	2.33	4.28	1.20	2.37	4.46	2.29	0.95	3.35	1.04	5.01	1.03	3.00	2.07
48	-	-	-	-	-	-	-	-	-	-	-	-	-	-

Legend: -, The content of cefquinome was less than the detection limit of the method (0.1 μg·kg^−1^).

## Data Availability

Data available in a publicly accessible repository.
